# An Inversion Model for Suspended Sediment Concentration Based on Hue Angle Optical Classification: A Case Study of the Coastal Waters in the Guangdong-Hong Kong-Macao Greater Bay Area

**DOI:** 10.3390/s25061728

**Published:** 2025-03-11

**Authors:** Junying Yang, Ruru Deng, Yiwei Ma, Jiayi Li, Yu Guo, Cong Lei

**Affiliations:** 1 School of Geography and Planning, Sun Yat-sen University, Guangzhou 510006, China; yangjy257@mail2.sysu.edu.cn (J.Y.); mayw8@mail2.sysu.edu.cn (Y.M.); lijy286@mail2.sysu.edu.cn (J.L.); guoy87@mail2.sysu.edu.cn (Y.G.); leic@mail2.sysu.edu.cn (C.L.); 2 Guangdong Engineering Research Center of Water Environment Remote Sensing Monitoring, Guangzhou 510006, China; 3 Southern Marine Science and Engineering Guangdong Laboratory (Zhuhai), Zhuhai 519000, China; 4 Guangdong Provincial Key Laboratory of Urbanization and Geo-Simulation, Guangzhou 510006, China

**Keywords:** suspended sediment concentration, hue angle, secondary-scattering approximation model, the GBA, the coastal waters

## Abstract

The Guangdong-Hong Kong-Macao Greater Bay Area (GBA) is one of the most urbanized and industrialized coastal regions in China, where intense human activities contribute to substantial terrestrial sediment discharge into the adjacent marine environment. However, complex hydrodynamic conditions and high spatiotemporal variability pose challenges for accurate suspended sediment concentration (SSC) retrieval. Developing water quality retrieval models based on different classifications of water bodies could enhance the accuracy of SSC inversion in coastal waters. Therefore, this study classified the coastal waters of the GBA into clear and turbid zones based on Hue angle α, and established retrieval models for SSC using a single-scattering approximation model for clear zones and a secondary-scattering approximation model for turbid zones based on radiative transfer processes. Model validation with in-situ data shows a coefficient of determination (R^2^) of 0.73, a root mean square error (RMSE) of 8.30, and a mean absolute percentage error (MAPE) of 42.00%. Spatial analysis further reveals higher SSC in the waters around Qi’ao Island in the Pearl River Estuary (PRE) and along the coastline of Guanghai Bay, identifying these two areas as priorities for attention. This study aims to offer valuable insights for SSC management in the coastal waters of the GBA.

## 1. Introduction

Suspended sediment concentration (SSC) is a critical parameter in the study of estuaries and coastal waters, playing a significant role in coastal marine ecosystems and geomorphological evolution [1,2]. Through scattering, refraction, and absorption of light, suspended sediments (SSs) markedly affect the optical properties of seawater [3]. High SSC increases water turbidity, reducing transparency and diminishing incident solar radiation. This, in turn, weakens phytoplankton photosynthesis, adversely impacting their growth and primary productivity [4]. This stresses marine ecosystems and directly influences critical global material transfer processes. Moreover, SSs act as key carriers for the migration and re-circulation of pollutants, as their particles possess a high specific surface area and strong adsorption capacity, enabling them to bind with a variety of pollutants, including heavy metals, organic contaminants, and nutrients [5,6]. This adsorption process facilitates the migration and diffusion of pollutants with suspended sediments or their settling and immobilization. Therefore, monitoring SSC is crucial for effective pollutant control in coastal waters.

Currently, SSC monitoring in coastal waters primarily relies on manual water sampling and buoy monitoring stations. However, manual sampling and buoy monitoring are labor- and cost-intensive, and they face particular challenges in capturing dynamic water quality changes effectively across large coastal areas [7]. In contrast, remote sensing technology overcomes the cost and timeliness limitations of traditional sampling methods, offering large-scale, periodic water quality monitoring for targeted water bodies, significantly reducing labor and time costs [8,9]. Satellite-based remote sensing retrieval algorithms for SSC are typically categorized into empirical, semi-analytical, and analytical models. Cai et al. developed a linear model using the HaiYang-1C (HY-1C) satellite to retrieve SSC in the Zhoushan offshore waters [10]. Ribeiro Marinho et al. developed a linear regression model using the near-infrared band of Sentinel-2 satellites to retrieve SSC in the Negro River, Amazon Basin [11]. Larson et al. used Landsat 8 OLI to map SSC within the Maumee River in Ohio, USA, at multiple depth intervals, applying simple linear least squares regression and three machine learning models to estimate SSC, respectively [12]. Empirical models are based on data-driven patterns, but their generalization capabilities are relatively limited. Zhao et al. retrieved SSC in the Bohai Bay of China using the QAA model [13]. Bernardo et al. improved IOP estimates using the re-parameterized model QAATRCS, enhancing the accuracy of a_t_, b_b_, and Kd estimates, and retrieved the predicted SSC using Kd(655) and a power fitting [14]. While semi-analytical models are critical, most studies have overlooked water turbidity and relied on single-scattering approximations. This simplification can introduce significant uncertainties in high-turbidity areas. Therefore, SSC models should account for multiple-scattering effects in moderate-to-high turbidity regions.

In addition to accounting for multiple scattering, water optical classification methods can effectively reduce uncertainties, thereby enhancing the generalization ability of models and improving computational efficiency, which is especially important for practical applications [15,16,17]. Currently, researchers primarily use methods such as single-band [18], band ratios [19], spectral indices [20], and water color indices [21] for water classification. The Forel–Ule index (FUI) is particularly effective in mitigating aerosol interference and the effects of sensor observation geometry, providing a stable threshold for optical classification [22]. This reduces the impact of external conditions and enhances the consistency of suspended sediment concentration (SSC) measurements across different sub-models [23]. However, FUI is a discrete classification of the hue angle, and its data fluctuation range at different levels shows significant variation. As a continuous parameter, the hue angle can overcome the limitations of FUI and offer a more refined expression of water color information.

This study focuses on the coastal waters of the Guangdong-Hong Kong-Macao Greater Bay Area (GBA). Optical classification of the coastal waters was performed based on the hue angle α. A single-scattering physical model based on radiative transfer processes was developed for the clear zone, while an approximate simplification method was applied to create an SSC second-scattering retrieval model for the turbid zone. This study also analyzes the spatial distribution of SSC in the coastal waters of the GBA.

## 2. Methodology

### 2.1. Study Area

**Figure 1:**
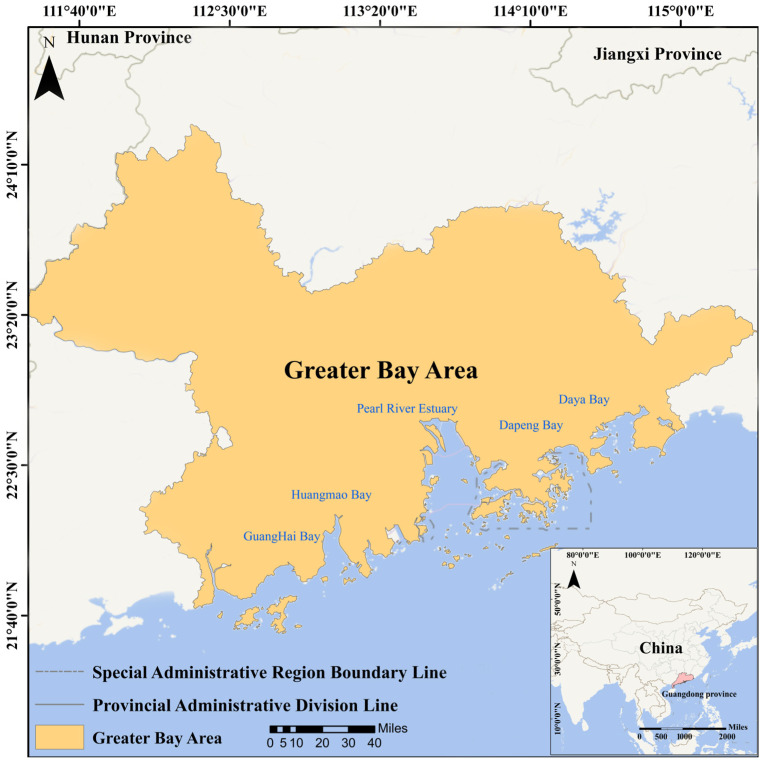
The location of the GBA.

The Guangdong-Hong Kong-Macao Greater Bay Area (GBA), located in southern China, is a major economic and urban cluster comprising nine cities in Guangdong Province: Guangzhou, Shenzhen, Zhuhai, Foshan, Dongguan, Jiangmen, Zhongshan, Huizhou, and Zhaoqing, along with the two Special Administrative Regions of Hong Kong and Macao. The GBA serves as a critical hub for economic growth, innovation, and trade within both China and the Asia-Pacific region. The coastal region primarily encompasses areas surrounding the Pearl River Estuary (PRE), as shown in Figure 1. To the right of the PRE are two semi-enclosed bays, Daya Bay (DYB) and Dapeng Bay (DPB), while to the left are Guanghai Bay and Huangmaohai, as illustrated in Figure 1. The hydrological characteristics of the coastal waters in the Guangdong-Hong Kong-Macao Greater Bay Area are complex and diverse, primarily influenced by the Pearl River runoff, tides, and monsoons. The Pearl River Estuary experiences an irregular semi-diurnal tide, with a tidal range of approximately 1–2 m, and strong tidal currents in narrow channels. Salinity gradually increases from the estuary to the open sea, with a significant influence of runoff during the summer, leading to an expansion of low-salinity areas. The region’s climate is classified as a subtropical monsoon climate, with an annual mean temperature ranging from 14 °C to 22 °C and annual precipitation ranging from 1200 mm to 2200 mm. Precipitation is highly seasonal, with approximately 70–85% occurring during the wet season from April to September, leading to significant seasonal variations in sediment transport. Suspended sediment concentrations are considerably higher during the wet season than in the dry season. The coastal waters of the GBA are crucial for regional economic growth and ecosystem conservation. Strengthening water quality monitoring and pollution management is essential for the sustainable environmental development of the region.

### 2.2. Study Data

Landsat 8 satellite data, a collaborative Earth observation product launched by the United States Geological Survey (USGS) and the National Aeronautics and Space Administration (NASA), have been available since 2013 as the eighth satellite in the Landsat series. Equipped with two imaging instruments—the Operational Land Imager (OLI) and the Thermal Infrared Sensor (TIRS)—Landsat 8 provides valuable remote sensing data. The OLI sensor includes nine spectral bands, incorporating the legacy bands for blue, green, red, near-infrared (NIR), and shortwave infrared (SWIR), inherited from previous Landsat missions, as well as additional deep blue and quality assurance bands. The deep blue band is specifically optimized for water body and coastal observations. Landsat 8 OLI offers a spatial resolution of 30 m for most bands and a scene width of 185 km, with a 16-day revisit cycle, making it highly suitable for surface coverage and temporal resolution requirements. The data from Landsat 8 are widely used in land cover classification, ecological monitoring, water quality assessment, agricultural management, and urban expansion studies. In water quality studies, the multispectral data from Landsat 8 can be applied to derive parameters such as suspended sediments, chlorophyll concentration, and colored dissolved organic matter (CDOM), all of which are crucial for understanding patterns of water environmental change.

Field sampling for spectra and water quality data was conducted by the research team in the Pearl River Estuary on 22 August and 25 September 2019, as well as on 5 November, 12 November, and 27 November 2020. SSCs were measured within 24 h of sampling at the China National Analytical Center (Guangzhou, China). Reflectance data were measured synchronously using a spectrometer (ASD FieldSpec3 spectroradiometer manufactured by the U.S. Company ASD, Falls Church, VA, USA), capable of detecting reflectance at 1 nm intervals over a range of 350 nm to 2500 nm. Additionally, water quality data collected from April to November 2020 by the Guangdong Provincial Department of Ecology and Environment (GPDEE), from 2019 to 2023 by the Hong Kong Environmental Protection Department (HKEPD), and from 2020 to 2023 by the Hydrological Bureau of Guangdong Province (HBGP) were included. A total of 5017 SSC data were collected (Table 1). The monitoring station locations are shown in Figure 1. After satellite and in-situ data matching, 165 satellite-ground data pairs were obtained, with SSCs ranging from 0.6 to 79 mg/L.

### 2.3. Landsat8 OLI Image Processing

**Figure 2:**
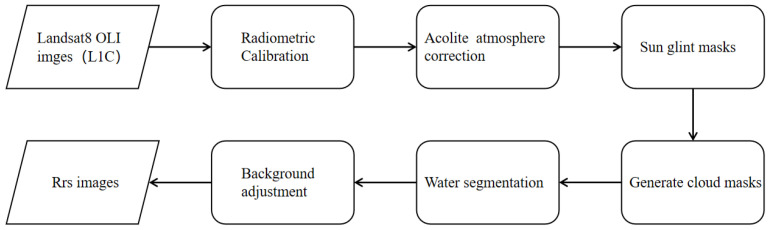
Landsat8 OLI image processing workflow.

All available Landsat 8 OLI Collection 2 L1C images with less than 10% cloud cover over the study area were obtained from the United States Geological Survey (USGS). The image processing workflow (illustrated in Figure 2) began with radiometric correction according to the official USGS guidelines. Atmospheric correction was applied using the Acolite method, as described in [24,25]. Sun glint masks used the Cox–Munk model [26], and cloud masks were conducted via a threshold-based extraction method. Water segmentation was performed using the Normalized Difference Water Index (NDWI). Finally, background adjustments were made to generate remote sensing reflectance (Rrs) imagery.

### 2.4. Water Optical Classification

The water classification method based on the hue angle follows the approach outlined by [27], with the specific steps as detailed below.

First, the visible bands of Landsat 8 OLI are used to derive tristimulus values based on the CIE-RGB conversion method:

(1) $$\begin{array}{cc} X & =2.7689*Rrs(R)+1.7517*Rrs(G)+1.1302*Rrs(B)\\ Y & =1.0000*Rrs(R)+4.5907*Rrs(G)+0.0601*Rrs(B)\\ Z & =0.0000*Rrs(R)+0.0565*Rrs(G)+5.5934*Rrs(B) \end{array}$$

In Formula (1), Rrs(R), Rrs(G), and Rrs(B) represent the red, green, and blue bands of Landsat 8 OLI, respectively, while X, Y, and Z correspond to the three tristimulus values in the CIE color space.

To calculate the CIE chromaticity coordinates (x, y), the tristimulus values X, Y, and Z are first normalized to the range of 0 to 1 using the following formulas:

(2) $$\begin{array}{cc} x & =\frac{X}{X+Y+Z}\\ y & =\frac{Y}{X+Y+Z} \end{array}$$

In Formula (2), x and y represent the chromaticity coordinates in the CIE color space.

The hue angle α can be calculated from the chromaticity coordinates (x,y) as follows:

(3) $$\alpha =arctan2(y-\frac{1}{3},x-\frac{1}{3})$$

In Formula (3), α is the hue angle, ranging from 0° to 360°, and arctan2 is the two-variable arctangent function.

The hue angle α is then subjected to delta correction to eliminate color discrepancies caused by the Landsat 8 OLI band settings, as detailed in the study [28].

### 2.5. Model Construction

In our previous study, a retrieval model based on single-scattering approximation was established [29], as shown in the formula (Formula (4)), with the relevant formulas and derivations provided in the Supplementary Materials. In this study, we further adopted an approximate simplification method to develop a second-scattering approximation model (Formula (5)) and introduced a hue angle-based optical classification method. When the hue angle is below a certain threshold, the water is classified as a clear zone, and a single-scattering approximation model is applied. When the hue angle exceeds this threshold, the water is considered a turbid zone, and a secondary-scattering model is used.

(4) $$R_{w}=\frac{1-\rho (\theta ,\theta^{\prime })}{2\mu cos\varphi^{\prime }}\frac{\beta_{w}+2\sum D_{i}c_{ib}\beta_{i}}{cos\varphi^{\prime }\mu (2\alpha_{w}+\beta_{w}+2\sum D_{i}(\alpha_{i}+c_{ib}\beta_{i}))}$$

(5) $$R_{w}=\frac{1-\rho (\theta -\theta^{\prime })}{2\mu cos\varphi^{\prime }}[\frac{\beta_{w}+2\sum D_{i}c_{ib}\beta_{i}}{cos\varphi^{\prime }\mu (2\alpha_{w}+\beta_{w}+2\sum D_{i}(\alpha_{i}+c_{ib}\beta_{i}))}+\frac{{\beta_{w}}^{2}+4\sum D_{i}c_{ib}(1-c_{ib}){\beta_{i}}^{2}}{16{\left[\alpha_{w}+\beta_{w}+\sum D_{i}(\alpha_{i}+\beta_{i})\right]}^{2}}]$$

### 2.6. Spatial Analysis

This study collected Landsat 8 imagery with less than 20% cloud cover from October to March between 2019 and 2023 to map the spatial distribution of SSC in the coastal waters of the GBA during the autumn–winter season. Due to Landsat 8’s relatively long revisit cycle, cloud cover tends to be higher from April to September, resulting in poorer inversion quality and introducing significant uncertainty in annual SSC spatial mapping. Therefore, this study focuses on mapping the SSC spatial distribution in the coastal waters of the GBA during the autumn–winter season. After collecting the imagery for each pass, SSC inversion was performed, and invalid pixels, such as background and cloud pixels, were excluded. The spatial distribution map of SSC was then generated by averaging the valid pixel values.

### 2.7. Accuracy Metrics

To evaluate the accuracy of the SSC inversion model for the GBA, three indicators were used: coefficient of determination (R^2^), root mean square error (RMSE), and the mean absolute percentage error (MAPE).

The formula for calculating R^2^ is

(6) $$\begin{array}{c} R^{2}=1-\frac{\sum {\left(y_{i}-f_{i}\right)}^{2}}{\sum {\left(y_{i}-Y\right)}^{2}} \end{array}$$

where “$R^{2}$” is the coefficient of determination, “$y_{i}$” is SSC at the validation point, “$f_{i}$” is the value retrieved from the inversion model, and “Y” is the mean SSC at the validation points.

The formula for calculating RMSE is

(7) $$\begin{array}{c} \mathrm{RMSE}=\sqrt{\frac{\sum {\left(y_{i}-f_{i}\right)}^{2}}{n}} \end{array}$$

where “n” is the sample size, “$y_{i}$” is SSC at the validation point, and “$f_{i}$” is the value retrieved from the inversion model.

The formula for calculating MAPE is

(8) $$\begin{array}{c} \mathrm{MAPE}=\frac{1}{n}\sum \left|y_{i}-f_{i}\right|*100\% \end{array}$$

## 3. Result

### 3.1. Comparison Between Hue Angle and SSC Values

**Figure 3:**
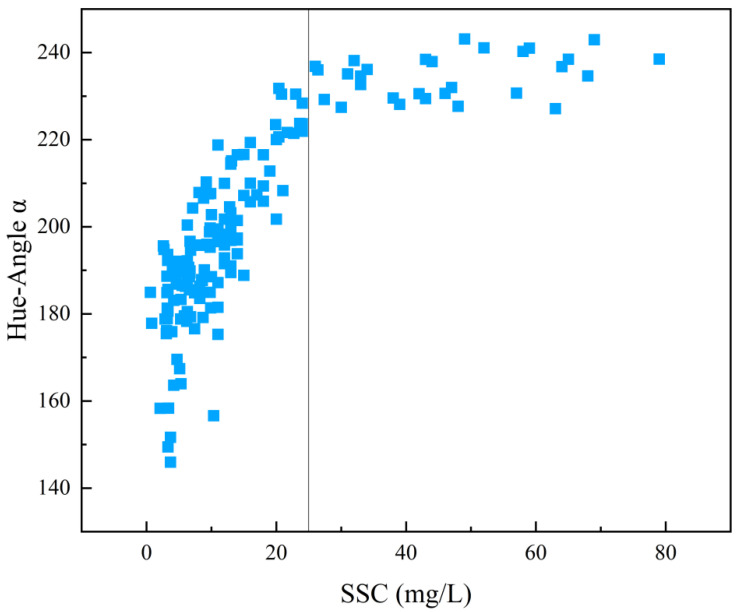
Relationship between hue angle and in-situ SSC.

**Figure 4:**
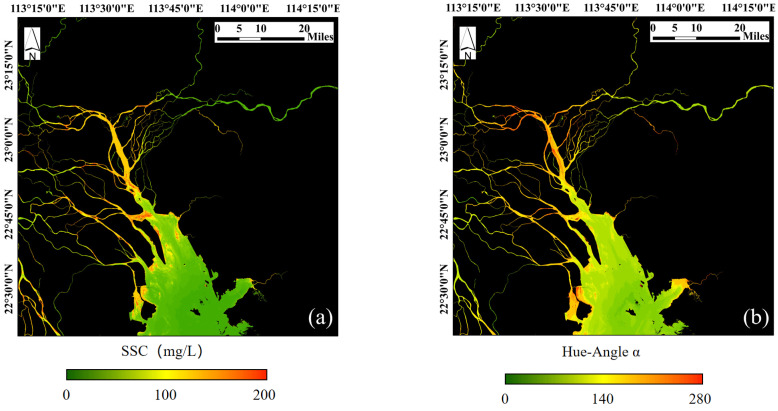
SSC retrieved from single-scattering model (**a**) on 19 January 2021 and hue angle α (**b**).

This section examined the relationship between hue angle and in-situ SSC values. The results reveal a significant linear correlation between hue angle and SSC in areas with low SSC, where the hue angle increases as SSC rises (Figure 3). However, in high SSC regions, further increases in SSC do not result in a significant rise in hue angle. Since the coastal waters of the GBA do not exhibit extremely high SSC levels compared to the Yellow River or Yangtze River basins, most areas maintain low to moderate SSC. Therefore, this study set an SSC threshold of 25 mg/L to distinguish between clear and turbid zones. The hue angle corresponding to 25 mg/L was calculated to be 231°, and this threshold was used to classify clear and turbid zones. Additionally, we mapped SSC (Figure 4a) and hue angle images (Figure 4b) for the PRE on 19 January 2021. Visual interpretation shows that the spatial distribution of SSC closely resembles that of hue angle. Based on the statistical and spatial relationship between SSC and hue angle, using hue angle to classify water regions in the GBA appears to be a reasonable approach.

### 3.2. Validation of the SSC Inversion Model

We developed a novel model based on the optical classification results derived from hue angle α. In this model, water bodies are classified into clear and turbid zones using a hue angle threshold of 231°. The model is expressed as follows:

(9) $$R_{w}=\frac{1-\rho (\theta -\theta^{\prime })}{2\mu cos\varphi^{\prime }}\frac{\beta_{w}+2\sum D_{i}c_{ib}\beta_{i}}{cos\varphi^{\prime }\mu (2\alpha_{w}+\beta_{w}+2\sum D_{i}(\alpha_{i}+c_{ib}\beta_{i}))},\;\;\;\;Angle<231{}^{\circ}\;R_{w}=\frac{1-\rho (\theta -\theta^{\prime })}{2\mu cos\varphi^{\prime }}\left[\frac{\beta_{w}+2\sum D_{i}c_{ib}\beta_{i}}{cos\varphi^{\prime }\mu (2\alpha_{w}+\beta_{w}+2\sum D_{i}\left(\alpha_{i}+c_{ib}\beta_{i}\right))}+\frac{{\beta_{w}}^{2}+4\sum D_{i}c_{ib}\left(1-c_{ib}\right){\beta_{i}}^{2}}{16{\left[\alpha_{w}+\beta_{w}+\sum D_{i}\left(\alpha_{i}+\beta_{i}\right)\right]}^{2}}\right],\;\;Angle>231{}^{\circ}$$

In Formula (9), α represents the absorption coefficient, β represents the scattering coefficient, $c_{b}$ is the proportion of backward-scattering during scattering, the subscript w represents water molecules, i represents components present in the water, and the concentration of each component is denoted as $D_{i}$. The angle between the sensor’s observation direction and the vertical direction is φ, the solar zenith angle is θ, the refraction angle of the incident light entering the water is $\theta^{\prime }$, and the refraction angle of the observation direction in the water is $\varphi^{\prime }$.

**Figure 5:**
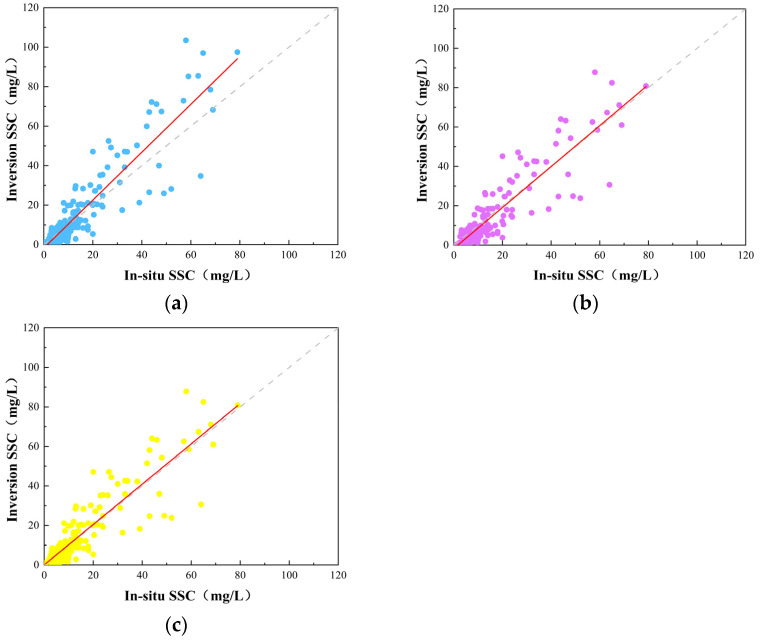
Accuracy assessment result. (**a**) Single-scattering approximation model. (**b**) Secondary-scattering approximation model. (**c**) Angle-SS model.

Based on the accuracy evaluation results, the R^2^ of the single-scattering approximation model for SSC retrieval is 0.60, with an RMSE of 10.11 and a MAPE of 44.09% (Table 2). The single-scattering approximation model tends to overestimate SSC in turbid zones, resulting in a relatively high R^2^ and RMSE. This overestimation occurs because the single-scattering approximation model neglects the actual occurrence of multiple scattering, which becomes more significant in high-SSC-concentration areas. The secondary-scattering approximation model and the Angle-SS model provide better estimation results compared to the single-scattering approximation model, with a notable improvement in R^2^, reaching 0.71 and 0.73 (Table 2), respectively. The RMSE decreases to 8.38 and 8.30, respectively. In high-SSC regions, the biased estimation seen in the single-scattering approximation model is reduced in both the secondary-scattering approximation and Angle-SS models, with the matching points aligning along the 1:1 line (Figure 5). Compared to the secondary-scattering approximation model, the MAPE of the Angle-SS model based on optical classification is reduced by 3.47%. This improvement is attributed to the superior estimation performance of the Angle-SS model in the clear zone.

### 3.3. Spatiotemporal Analysis of SSC in the Greater Bay Area

**Figure 6:**
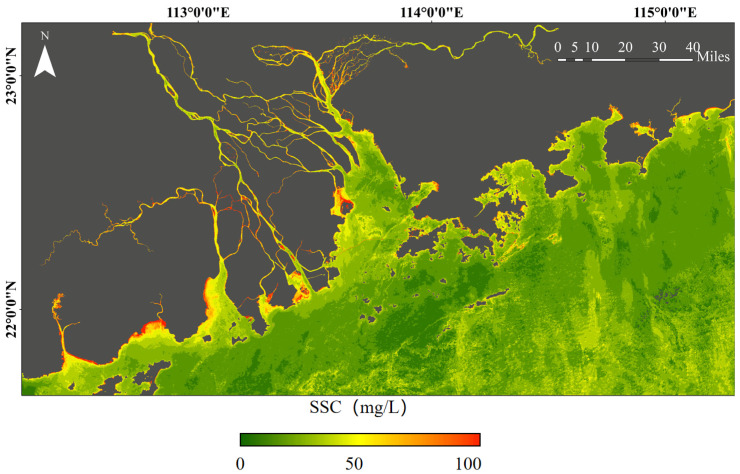
Spatial distribution of SSC in the coastal waters of the GBA.

**Figure 7:**
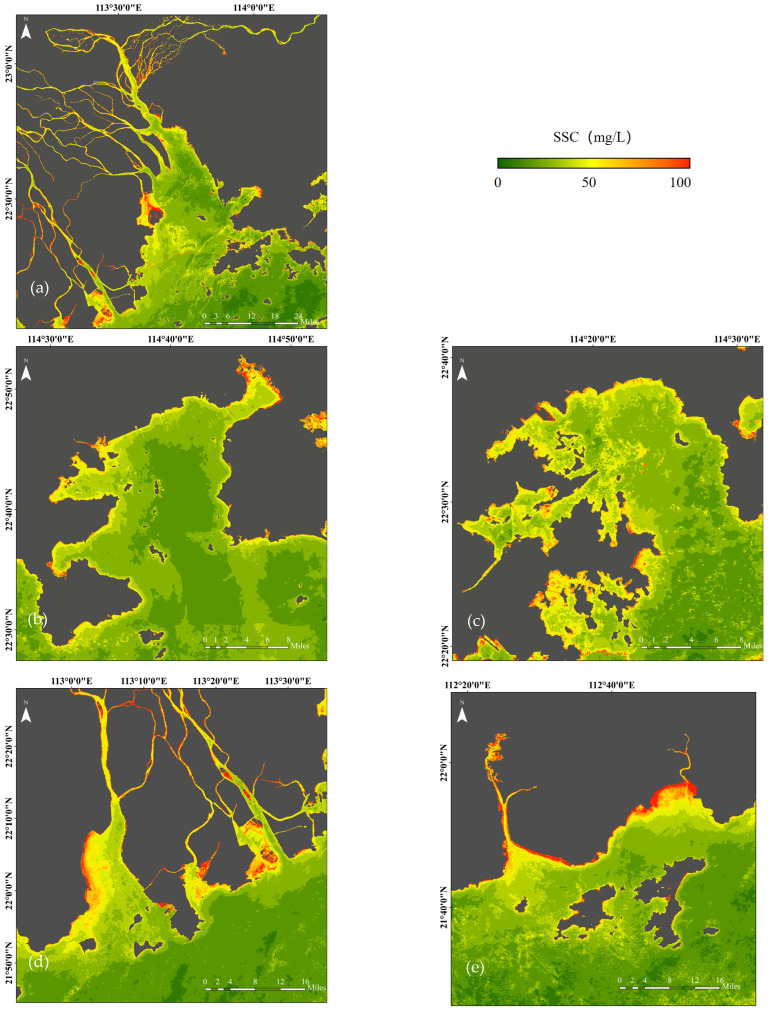
Spatial distribution map of SSC in the PRE (**a**), Daya Bay (**b**), Dapeng Bay (**c**), Huangmao Bay (**d**), and Guanghai Bay (**e**).

The spatial distribution of SSC in the GBA during the autumn–winter season shows a gradual decrease from nearshore to offshore, from southwest to northeast, which follows the general pattern of terrestrial input (Figure 6). This is primarily due to the influence of the winter northeast monsoon and tidal movements, which cause suspended sediments to accumulate mainly in the southwestern nearshore areas of the GBA. High SSC values were observed in the waters along the southwestern coasts of the Pearl River Estuary (PRE) (Figure 7a), Huangmao Bay (Figure 7d), and Guanghai Bay (Figure 7e), where a large amount of suspended sediment carried by the West River enters the estuary. Suspended sediments are easily trapped in lateral estuaries and shallow areas, leading to higher SSC in these regions [30]. In contrast, the eastern coastal waters of the GBA exhibit relatively lower SSC values, as the East River has lower SSC, and many small streams flow into the main river mouth, resulting in clearer waters near Daya Bay (Figure 7b) and Dapeng Bay (Figure 7c) in Shenzhen. However, scattered areas of high SSC are also found along the coasts of Daya Bay and Dapeng Bay, which is closely related to human activities near the Shenzhen coastline [31].

## 4. Discussion

### 4.1. Comparison of SSC Inversion Models

**Figure 8:**
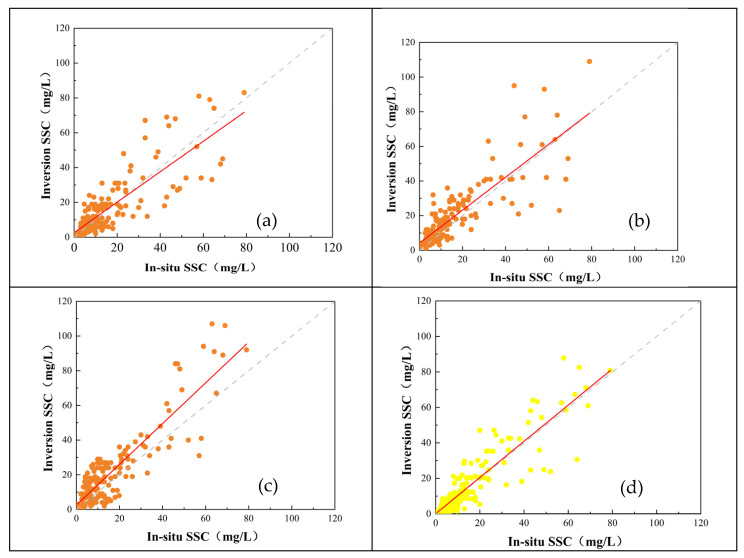
Accuracy assessment result of Yu model (**a**), Cao model (**b**), Yue model (**c**), and Angle-SS model (**d**).

In this study, we also compare several advanced remote sensing models of other authors for SSC retrieval, using RMSE and MAPE as evaluation metrics. The performance of the Angle-SS model and the Yue model [32] outperformed that of the Yu model [33] and the Cao model [31] (Figure 8). The RMSE and MAPE of the Yu model are 11.55 and 79.57%, respectively (Table 3). The relatively higher MAPE compared to other models may be attributed to the fact that its design is more suited for SSC retrieval in inland waters. Therefore, we selected the Cao model, which has been applied to the PRE, with an RMSE of 10.65 and a MAPE of 60.39%. The Cao model used only the near-infrared band as input, which might have contributed to the relatively higher RMSE. However, the Yue model achieved RMSE and MAPE values of 9.85 and 52.36%, respectively, demonstrating strong performance. This suggests that water optical classification is a recommended approach to enhance model performance.

### 4.2. Uncertainty Analysis

The spectral signal of the water body received by sensors is often influenced by various factors such as the atmosphere, watershed environment, and water depth. In particular, atmospheric interference is significant, with over 90% of the radiation signal detected by sensors originating from atmospheric contributions, while information related to the water body accounts for less than 10% of the signal [34]. Therefore, accurate atmospheric correction is the most important preprocessing step for SSC inversion [35]. The shadows of clouds, ships, and riverbanks are also difficult to completely eliminate [36,37]. The reliability of satellite sensors, differences between sensors, and the mixed spectral information in certain marine areas can all lead to discrepancies in SSC mapping. This will be a key research direction for future SSC inversion studies and should focus more on the quantitative analysis of uncertainties in these aspects.

## 5. Conclusions

Water quality plays a critical role in coastal ocean management and the ecological development of coastal regions. As a significant indicator of pollutants, regular monitoring of suspended sediment concentration (SSC) provides essential insights into the health of coastal water environments. In this study, the coastal waters of the Guangdong-Hong Kong-Macao Greater Bay Area (GBA) were classified into two zones: clear zones and turbid zones, using the hue angle (α). Remote sensing inversion models for SSC were developed by employing a single-scattering approximation model for clear waters and a secondary-scattering approximation model for turbid waters. Validation using in-situ SSC data and comparison with models from other researchers showed that the SS model achieved better predictive performance. Spatial analysis revealed higher SSC levels in the waters around Qi’ao Island in the Pearl River Estuary (PRE) and along the coastline of Guanghai Bay. These two areas should be prioritized for monitoring and management. Consequently, future efforts should focus on further analyzing the pollution sources in these key regions.

## Figures and Tables

**Table 1:** SSC statistical summary.

Dataset	Number	Max (mg/L)	Min (mg/L)	Mean (mg/L)
Sampling data	43	36	0.6	13.71
GPDEE data	654	263.1	0.3	9.36
HKEPD data	4234	360	0.5	8.15
HBGP	86	173	1.2	10.08
Total	5017	360	0.3	8.65

**Table 2:** Accuracy evaluation metrics of the three models.

Model	R^2^	RMSE	MAPE
Single-Scattering	0.60	10.11	44.09%
Secondary-Scattering	0.71	8.38	45.47%
Angle-SS	0.73	8.30	42.00%

**Table 3:** SSC Inversion Model Comparison.

Model	Model Form	RMSE	MAPE
Yu [33]	SSC = 0.8079 × BRed/BGreen + 23.062 × BNIR + 0.6258	11.55	79.57%
Cao [31]	SSC = 3.501 × e^4.317^ × Rrs	10.65	60.39%
Yue [32]	lnSSCclass1 = 65.011 × Rrs665 − 2.243 × Rrs740/Rrs665 + 4.09 × Rrs705/Rrs665 − 0.449, MCI ≤ 0.0016 lnSSCclass1 = −30.941 × Rrs740 − 2.667 × Rrs740/Rrs492 + 3.224, MCI ≤ 0.0016 (MCI: maximum chlorophyll index)	9.85	52.36%
This study	-	8.30	42.00%

## Data Availability

All data are contained within the article.

## Supplementary Materials

The following supporting information can be downloaded at: https://www.mdpi.com/article/10.3390/s25061728/s1, Figure S1. The single-scattering underwater light path diagram. Figure S2. The secondary-scattering underwater light path diagram. reprsents the incident direction and the observation direction respectively. The single-scattering light originates from above the water layer (a). The single-scattering light originates from below the water layer (b).
