# Supplementary material for: An Inversion Model for Suspended Sediment Concentration Based on Hue Angle Optical Classification: A Case Study of the Coastal Waters in the Guangdong-Hong Kong-Macao Greater Bay Area
Source: Sensors (Basel). 2025 Mar 11;25(6):1728. doi: 10.3390/s25061728 (PMC11946726; doi:10.3390/s25061728)
Supplement: Supplementary file 1 [file sensors-25-01728-s001.zip › sensors-3475300-supplementary.pdf]

Title:

An Inversion Model for Suspended Sediment Concentration Based on Hue-Angle Optical Classification: A Case Study of the Coastal Waters in the Guangdong-Hong Kong-Macao Greater Bay

Authors:

Junying Yang[1,2,3,4], Ruru Deng[1,2,3,4*], Yiwei Ma[1,2,3], Jiayi Li[1,2,3,4], Yu Guo[1,2,3,4], Cong Lei[1,2,3,4]

Supplementary material:

1. Single Scattering Approximation Model

As shown in Figure. S1, assume that the angle between the sensor's observation direction and the vertical direction is φ, the solar zenith angle is θ, the refraction angle of the incident light beam entering the water is θ', and the refraction angle of the observation direction in the water is φ'. The reflectance above the water surface consists of scattered light in the water L_s and reflected light from the water bottom L_b.

$$L_{w1}(\varphi) = L_{s1}(\varphi') + L_b(\varphi') \quad \text{(Sf1)}$$

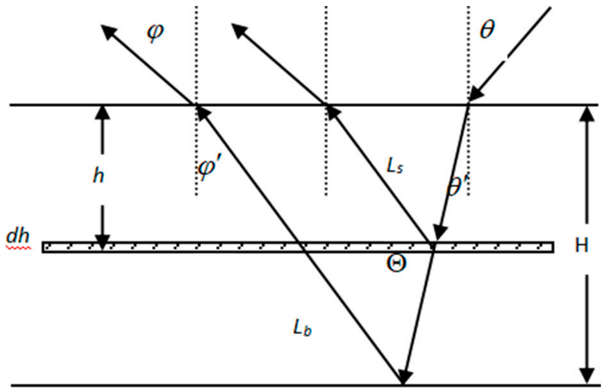

Figure. S1 The single-scattering underwater light path diagram

Calculated according to Snell's law：

$$\sin(\theta') = \sin(\theta) \,/\, n_w$$
$$\sin(\varphi') = \sin(\varphi) \,/\, n_w \quad \text{(Sf2)}$$

In Formula (Sf2), n_w represents the refractive index of water.

Due to the complexity of obtaining the scattering phase function, this study divides scattering into the upper hemisphere (back-scattering) and lower hemisphere (forward-scattering), assuming uniform scattering intensity in all directions within each hemisphere. This means that light propagates downward as diffuse radiation, with forward-scattering by waterborne particles assumed to be significantly greater than back-scattering. If the downward irradiance at depth h is $E_d(h)$, the increment in downward irradiance for a thin water layer with thickness dh at a depth h is given by:

$$dE_d(h) = -E_d(h)k dh/cos\theta' + E_d(h)b_f dh/cos\theta' \quad \text{(Sf3)}$$

In the Formula (Sf3), k represents the extinction coefficient of the water body, calculated by:

$$k = \alpha + b_f + b_b \quad \text{(Sf4)}$$

In the Formula (Sf4), α represents the total absorption coefficient, b_f represents

the forward-scattering coefficient of the water body. The Formula (Sf4) can be expressed as:

$$dE_d(h) = -E_d(h)(a + b_b)dh/cos\theta' \quad \text{(Sf5)}$$

Thus, the downward irradiance at depth h can be expressed as:

$$E_d(h) = E_d(0)e^{-(a+b_b)h/cos\theta'} \quad \text{(Sf6)}$$

The relationship between the radiance in a given direction and the incident irradiance can be expressed as:

$$L = \frac{E_0 w}{4\pi} p(\Theta) \quad \text{(Sf7)}$$

In the Formula (Sf7), w is the single-scattering albedo, $p(\Theta)$ is scattering phase function.

Since the forward and backward scattering are equal in all directions, the scattering phase function is not considered, and the conversion between irradiance and radiance is performed only within the 2π hemisphere. If w_b represents back-scattering coefficient, the radiance in the downward direction can be calculated as:

$$L_{u1} = \frac{E_0 w_b}{2\pi} \quad \text{(Sf8)}$$

The increment $dL_{s1}'(\varphi', h)$ of water body scattering radiance in the direction φ' at depth h can be expressed as:

$$dL_{s1}'(\varphi', h) = \frac{E_d(h) b_b dh/cos\varphi'}{2\pi} \quad \text{(Sf9)}$$

During the upward transmission process, the increment $dL_{s1}'(\varphi', h)$ of water body scattering radiance undergoes another attenuation, and can be expressed as:

$$dL_{s1}'(\varphi', h) = \frac{E_d(h) b_b dh / cos\varphi'}{2\pi}$$
$$dL_{s1}'(\varphi', h) = \frac{E_d(0) b_b dh / cos\varphi'}{2\pi} \quad \text{(Sf10)}$$

Let μ equal $\frac{1}{cos\theta'} + \frac{1}{cos\varphi'}$, then the total intensity of the direct scattering light in the entire water layer is:

$$L_{s1}(\varphi') = \int_0^H dL_{s1}'(\varphi', h) = \frac{E_d(0)}{2\pi\mu cos\varphi'} \quad \text{(Sf11)}$$

Based on the physical mechanism of water body reflection, the contribution of the bottom reflection light to the water-leave reflectance can be calculated as:

$$L_b(\varphi') = \frac{E_d(H) R_b}{\pi} \quad \text{(Sf12)}$$

In the Formula (Sf12), $R_b$is the bottom reflectance. When the water depth (H) approaches infinity, $L_b(\varphi')$ tends to zero and can be neglected.

When considering only single-scattering, the water-leave reflectance can be calculated as:

$$R_w = \frac{1-\rho(\theta,\theta')}{2\mu cos\varphi'} \frac{\beta_w + 2\sum D_i\, c_{ib}\beta_i}{cos\varphi'\mu(2\alpha_w + \beta_w + 2\sum D_i(\alpha_i + c_{ib}\beta_i))} \quad \text{(Sf13)}$$

In the Formula (Sf13), c_b is the proportion of backward-scattering during scattering, the subscriptwrepresents water molecules, irepresents other components present in the water, and the concentration of each component is denoted as D_i.

2. Secondary scattering approximation model

Secondary-scattering refers to the phenomenon where the scattered light from primary scattering is scattered again by particles in the water. If a given scattering event is divided into two components: forward-scattering(hemispherical) and

backward-scattering(hemispherical), represented by w_f and w_b as their respective scattering coefficients, and then at a water depth h, the secondary scattering received by the sensor can be composed of two parts. One part is the secondary backward-scattering(denoted asE_{2b}) of the single-forward-scattering light (denoted as E_{1f}) occurring above the water layer (Figure. S2a). The other part is the secondary-forward-scattering(denoted asE_{2f}) of the single-backward-scattered light (denoted asE_{1b}) occurring above the water layer (Figure. S2b).

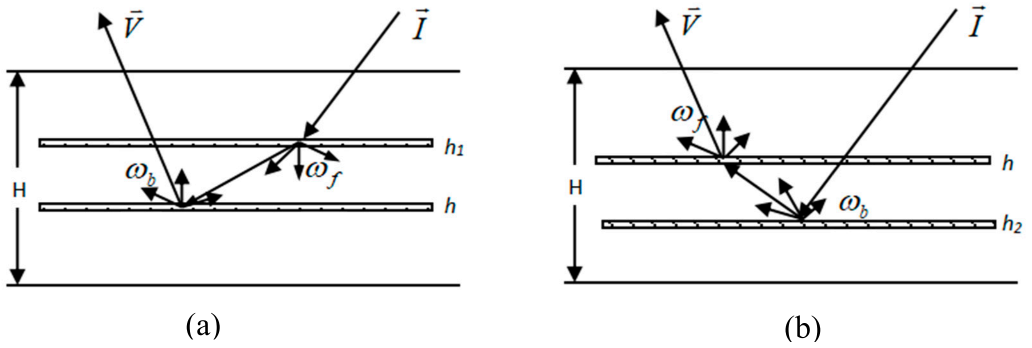

Figure. S2 The secondary-scattering underwater light path diagram. $\vec{I}$、$\vec{V}$ reprsents the incident direction and the observation direction respectively.The single-scattering light originates from above the water layer(a). The single-scattering light originates from below the water layer(b).

For a homogeneous water body, the optical thickness τ and transmittance of the water T can be calculated as follows:

$$\tau(h) = kh$$
$$T(h) = e^{-\tau(h)} = e^{kh} \quad \text{(Sf14)}$$

In the Formula (Sf14), k is the extinction coefficient.

Similar to the single-scattering model, the incident light intensity at the water surface can be calculated as:

$$E_d = E_0$$ $- \rho(\theta,\theta'))$	(Sf15)

Given that single-scattering light originates from all directions, the effect of angle on the transmission path is disregarded here. The single-forward-scattered light from a thin water layer at depth h_1 reaching a thin water layer at depth h can be expressed as:

$$E_{1f} = \int_0^h E_d b_f T(h_1) T(h - h_1) dT(h_1) = E_d b_f h e^{-kh}$$	(Sf17)

The secondary-scattering light formed by the re-scattering of E_{1f}undergoes further attenuation as it propagates out of the water surface. Therefore, for an entire water layer of depth H, the intensity of the secondary-backward-scattered light can be expressed as:

$$E_{2b} = \int_0^H E_{1f} b_b T(h) dh$$	(Sf18)

Similarly, the single-backward-scattering light from a thin water layer at depth h_2 reaching a thin water layer at depth h can be expressed as:

$$E_{1b} = \int_0^H E_d b_b T(h_2) T(h_2 - h) dT(h_2) = \frac{E_d b_b}{2k} (e^{-kh} - e^{-kh-2kH})$$	(Sf19)

The secondary-forward-scattering light intensity for the entire water layer of depth H can be expressed as:

$$E_{2f} = \int_0^H E_{1b} b_f T(h) dh$$	(Sf20)

Therefore, the secondary-scattering light for the entire water layer of depth H can be calculated as:

$$E_2 = E_{2f} + E_{2b} = \int_0^H T(h)(E_{1b} b_f + E_{1f} b_b) dh = \frac{E_d b_b b_f}{2k^2}(1 - e^{-2kh} \ \ - 2kHe^{-2kH} \ ) \quad \text{(Sf21)}$$

Since the incident light comes from all directions, the impact of the scattering phase function becomes negligible, and the scattered light intensity can be considered uniform in all spatial directions. Thus, the contribution of secondary-scattering to the water-leaving reflectance in the signal received by the sensor can be expressed as:

$$R_2 \approx \frac{E_2}{4\pi} \frac{\pi}{E_0 cos\theta} =$$

$$- e^{-2kh} \ \ - 2kHe^{-2kH} \ ) \quad \text{(Sf22)}$$

When the water depth approaches infinity, the function($f(H) = (1 - e^{-2kh} \ \ - 2kHe^{-2kH} \ )$) tends towards 1($\lim_{H \to \infty} f(H) = 1$). R_2 can be calculated as:

$$R_2 \approx$$

$$= \frac{(1 - \rho(\theta,\theta'))c_b(1 - c_b)\beta^2}{8(\alpha + \beta)^2} \quad \text{(Sf23)}$$

In the Formula (Sf23), c_b is the proportion of backward-scattering during scattering.

When considering secondary-scattering, the water-leaving reflectance is the sum of the single-scattering and secondary-scattering contributions. This can be expressed as:

$$R_{wo} =$$

$$+ R_2 = \frac{(1 - \rho(\theta,\theta'))}{2}[\frac{c_b \beta}{cos\varphi' \mu(\alpha + c_b \beta)^2} + \frac{c_b(1 - c_b)\beta^2}{4(\alpha + \beta)^2}] \quad \text{(Sf24)}$$

Since the absorption and scattering coefficients of the water body are the algebraic sums of the absorption and scattering coefficients of water molecules and

other components in the water, the subscript w represents water molecules, andirepresents other components present in the water. The concentration of each component is denoted as D_i(Formula(Sf25)).

$\beta =$ $+\sum D_i\, \beta_i$ $\alpha =$ $+\sum D_i\, \alpha_i$	(Sf25)

Therefore, Formula (Sf24) can be rewritten as:

$R_w = \frac{1-\rho(\theta-\theta')}{2\mu cos\varphi'}[\frac{\beta_w + 2\sum D_i\, c_{ib}\beta_i}{cos\varphi'\mu(2\alpha_w+\beta_w+2\sum D_i(\alpha_i+c_{ib}\beta_i))}+\frac{{\beta_w}^2+4\sum D_i c_{ib}(1-c_{ib})\,{\beta_i}^2}{16[\alpha_w+\beta_w+\sum D_i\,(\alpha_i+\beta_i)]^2}]$	(Sf26)
